# Low confidence in systematic reviews of digital interventions for physical activity promotion

**DOI:** 10.1093/eurpub/ckac129.301

**Published:** 2022-10-25

**Authors:** K Matthias, L Mergenthal, KK De Santis

**Affiliations:** 1 Faculty of Electrical Engineering and Computer Science, University of Applied Sciences Stralsund, Stralsund, Germany; 2 Department of Prevention and Evaluation, Leibniz Institute - BIPS, Bremen, Germany

## Abstract

**Background:**

The field of digital public health aims to promote and improve the health of people and communities through the application of digital interventions (DIs). Systematic reviews (SRs) show that many DIs target physical activity (PA) promotion. ‘A Measurement Tool to Assess Systematic Reviews’ (AMSTAR2) is a valid and moderately reliable appraisal tool with 16 items that can be used to derive an overall confidence rating (OCR: high, moderate, low or critically low) in the results of a SR. This cross-sectional study aimed to appraise SRs of DIs for PA promotion.

**Methods:**

This study using 30 SRs was embedded within a scoping review with a published protocol (doi:10.2196/35332). Following electronic searches in 03/2021 in MEDLINE, PsycINFO, and CINAHL two authors independently selected and appraised the SRs. AMSTAR2 appraisal outcomes were expressed as (1) OCRs according to the AMSTAR2 scoring guidelines and (2) as percentage scores (fulfilled items/16 items). Appraisal outcomes were assessed using descriptive statistics.

**Results:**

The 30 SRs were published in 2007-2021 and included 5-60 primary studies. Most SRs (27/30) received ‘critically low’ OCR, while ‘low’ (2/30), ‘moderate’ (1/30) or ‘high’ (0/30) OCRs were seldom. The 21 SRs published after AMSTAR2 implementation in 2018 onwards received higher percentage scores (mean±SD of 53±15%) compared to 9 SRs published before 2018 (44±13%). In addition, the 11 SR with a protocol received higher percentage scores (59±13%) compared to 19 SRs without a protocol (45±15%) and had fewer critical weaknesses (median: 2 vs. 4).

**Conclusions:**

High quality SRs are needed for making public health decisions. AMSTAR2 assigns mostly low and critically low OCR to SRs of DIs for PA promotion. These SRs should not be relied on as a source of accurate evidence. Our results show that SR reporting guidelines need to be better followed.

**Key messages:**

• According to AMSTAR2 the overall methodological quality of systematic reviews of digital interventions for physical activity promotion needs improvement.

• Better adherence to established reporting guidelines for systematic reviews in public health context is needed.

